# Resequencing and genome-wide association studies of autotetraploid potato

**DOI:** 10.1186/s43897-022-00027-y

**Published:** 2022-02-10

**Authors:** Feng Zhang, Li Qu, Yincong Gu, Zhi-Hong Xu, Hong-Wei Xue

**Affiliations:** 1 College of Agronomy, Gansu Provincial Key Laboratory of Aridland Crop Science, Gansu Key Laboratory of Crop Improvement & Germplasm Enhancement, Gansu Agricultural University, Lanzhou, 730070 China; 2 Shanghai Collaborative Innovation Center of Agri-Seeds, Joint Center for Single Cell Biology, School of Agriculture and Biology, Shanghai Jiao Tong University, Shanghai, 200240 China; 3 Shanghai OEbiotech, Shanghai, 201210 China; 4 National Key Laboratory of Plant Molecular Genetics, CAS Center for Excellence in Molecular Plant Sciences, Chinese Academy of Sciences, Shanghai, 200032 China

**Keywords:** *Solanum tuberosum* (potato), Autotetraploid cultivars, Genome-wide association study (GWAS), Domestication, Genome, Resequencing

## Abstract

Potato is the fourth most important food crop in the world. Although with a long history for breeding approaches, genomic information and association between genes and agronomic traits remain largely unknown particularly in autotetraploid potato cultivars, which limit the molecular breeding progression. By resequencing the genome of 108 main cultivar potato accessions with rich genetic diversity and population structure from International Potato Center, with approximate 20-fold coverage, we revealed more than 27 million Single Nucleotide Polymorphisms and ~ 3 million Insertion and Deletions with high quality and accuracy. Domestication analysis and genome-wide association studies (GWAS) identified candidate loci related to photoperiodic flowering time and temperature sensitivity as well as disease resistance, providing informative insights into the selection and domestication of cultivar potato. In addition, GWAS with GWASploy for 25 agronomic traits identified candidate loci by association signals, especially those related to tuber size, small-sized tuber weight and tuber thickness that was also validated by transcriptome analysis. Our study provides a valuable resource that facilitates the elucidation of domestication process as well as the genetic studies and agronomic improvement of autotetraploid potato.

**Supplementary Information:**

The online version contains supplementary material available at 10.1186/s43897-022-00027-y.

## Core

Seletive interval analysis combined GWAS of autotetraploid potato identified candidate loci under domestication and associated with agronomic traits, providing informative insights for elucidating  the domestication history and selective process of cultivar potato and facilitating the genetic studies and agronomic improvement of autotetraploid cultivar potato.

## Introduction

Potato (*Solanum tuberosum L.*) is the predominant vegetable crop in the global food system and also a critical food crop (Birch et al. 2012). Cultivated potatoes with autotetraploid inheritance have been domesticated from diploid wild species native to the Andes of Southern Peru (Spooner et al. 2005), and improved by domestication and modern breeding in many aspects including yield, quality and disease resistance. Consumer acceptance and distinct environmental conditions are the main reason for constrained spread of potatoes, and the domestication dispersal of crops outside of their native range need extensive adaptation to the new environments moved along latitudinal gradients (Diamond, 2002; Shennan et al. 2013). Elucidating the selective gene loci will surely help to understand the domestication of potato and relation between climate and human selection. However, the selective genes and domestication histories for cultivars are poorly understood yet.

Traditional breeding of potato is often faced with various difficulties. Considering the long breeding cycles and low selection efficiency, there is an urgent need to develop molecular approaches to facilitate the breeding efficiency. Although several molecular marker techniques including simple sequence repeat (SSR) and Expressed sequence tag-SSR (EST-SSR) have been applied to assess the genetic diversity, germplasm identification and population structure (Zhu et al. 2008; Rosyara et al. 2016; Zhao et al. 2017), however, the complexity of autotetraploid genome and insufficient genomic information are still big obstacles to functional research and molecular breeding (Levy and Veilleux, 2007). Autotetraploid cultivars are grown most widely all over the world and also the main materials used for cross breeding (Zhang et al. 2019), deciphering the genome sequence and analysis of trait diversity and genomic association is indispensable.

Several studies have sequenced some representative potato samples and mainly analyzed the sequence diversity to reveal the complex evolutionary history of adaptation to environments (Gutaker et al. 2019) and impact of domestication on genome diversity, and identified key loci selected for cultivation (Hardigan et al. 2017) or some molecular markers (Li et al. 2018). Recent advances in next-generation sequencing (NGS) and increasing capacity of computational analyses of massive data enabled us to comprehensively analyze the whole genome of autotetraploid potato cultivars with whole-genome sequencing (WGS). Indeed, genome-wide association study (GWAS) has been applied to discover causal variants for complex traits effectively with high resolution at genome level comparing with the traditional linkage mapping strategy (Zhu et al. 2008). Combination of WGS and GWAS has been shown as an effective method for identifying genome-phenotype associations in many crop species including rice (Huang et al. 2012), sorghum (Tao et al. 2020) and maize (Li et al. 2019), which provides informative genomic resources for functional studies. Nevertheless, the study is still very preliminary in potato especially with combination of WGS and GWAS.

The GWAS of autotetraploid potato is rare and difficult, mainly due to the lack of genomic information and accurate assembled sequence of autotetraploid potato as a reference. Fortunately, construction of a chromosome-scale long-read reference genome assembly enabled the GWAS of autotetraploid potato (Pham et al. 2020), which is an updated version of the DM1–3516 R44 genome sequence, a doubled monoploid clone of *S. tuberosum* Group Phureja using a whole-genome shotgun sequencing approach with short-read sequence data (Xu et al. 2011). Hence combination of WGS and GWAS as well as a chromosome-scale long-read reference genome make it possible to explore more genomic resources for cultivated autotetraploid potato.

In this study, we resequenced 108 core cultivated germplasms of autotetraploid potato from International Potato Center by whole-genome sequencing and revealed the loci associated with the rich genetic diversity by analyzing 25 agronomic traits with GWASploy analysis. One hundred and thirty-eight high-confidence selective sweeps comprising 54 predicted genes were dissected by domestication analysis, which is beneficial for investigating the diversity and domestication of cultivated potato. In addition, large scale GWAS studies identified 50 candidate loci associating with 15 agronomic traits. These results provide helpful and important genomic resources of cultivated potato and significantly contribute the potato biology and breeding approaches.

## Results

### Whole-genome sequencing of 108 CIP and identification of SNPs and InDels

In this study, 108 high-generation accessions from International Potato Center (designated as CIP accessions, Table S1) were applied for whole-genome sequencing and further analysis. Collected samples were used to construct libraries and genotyped with approximate 20-fold-coverage genome sequencing using a barcoded multiplex sequencing approach on the Illumina HiSeq PE (Paired-End) 150 base pairs (bp). Raw paired-end reads were trimmed and filtered to obtain the high-quality clean data. Trimmed reads were mapped to the *S. tuberosum* group Phureja DM reference genome (Pham et al. 2020; Xu et al. 2011) for alignment, and GATK (McKenna et al. 2010) was used to detect the population SNP (Single Nucleotide Polymorphisms) and InDel (Insertion and Deletion) (Table S2). After filtering and screening, the high quality and accuracy population SNPs and InDels were finally annotated and analyzed.

We finally identified 27,565,997 SNPs and 2,961,770 InDels by analyzing these CIP accessions, which lays a good foundation for the selective interval analysis and GWAS analysis. A total of 1,565,451 SNPs (4.21%) and 64,080 (1.37%) InDels were located in the coding regions, among which 60,265 SNPs or InDels showed potentially significant effects, including 21,694 SNPs (0.058%) that may affect 2329 genes by causing start codon changes, premature stop codons or elongated transcripts, and 38,571 InDels (0.832%) that may lead to a frameshift in 11,132 annotated genes (Table S3).

Previously, 67 genotypes including 20 Wild diploid species, 20 South American landraces, 23 North American cultivars and 4 Outgroups were resequenced (Hardigan et al. 2017), which were used to capture a broad extent of genome variation and their progenitors. We downloaded the resequencing data of these 67 accessions (NCBI database under PRJNA378971, Hardigan et al. 2017) and reanalyzed the 62 accessions of them together with the 108 CIP accessions, with a total of 170 accessions (5 of previous analyzed cultivars were included in CIP accessions for deep sequencing). After mapping with the reference genome (DM v6.1, Pham et al. 2020), we identified 22,003,848 SNPs and 1,878,036 InDels with an average of 25.88 SNPs and 2.16 InDels per kb. Detailed analysis showed that a total of 1,538,176 SNPs (5.06%) and 52,376 (1.75%) InDels were located in the coding regions, among which 47,533 are potentially being crucial including 19,007 SNPs (0.06%) affect 3074 genes by causing start codon changes, premature stop codons or elongated transcripts, and 30,552 (1.02%) InDels lead to a frameshift in 12,301 annotated genes (Table S4). Considering there are wild diploid species in the analyzed 170 accessions, these data will help the further domestication and selection analysis.

### Population structure divergence revealed that CIP accessions are typical cultivated autotetraploid potato

**Fig. 1:**
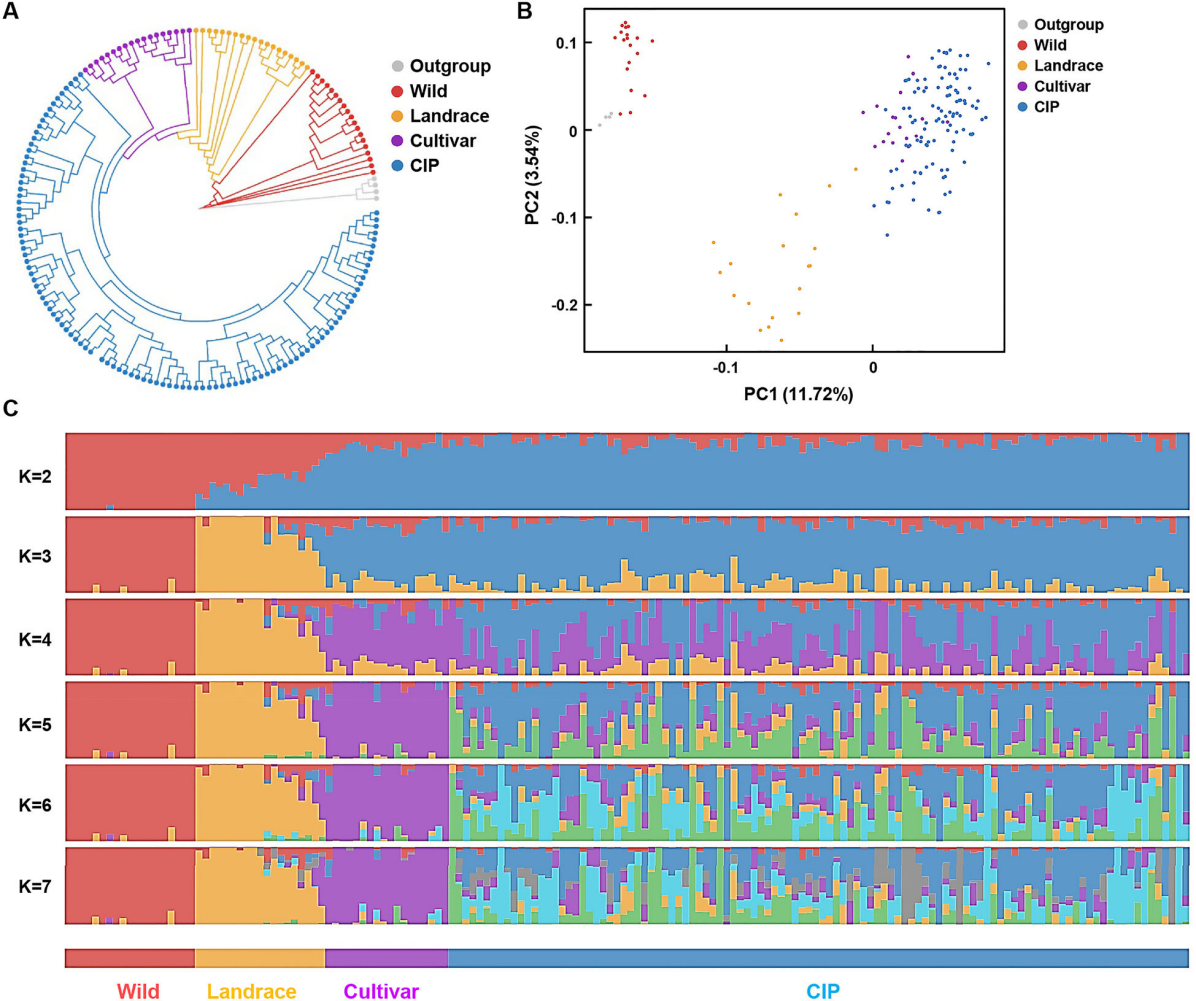
Phylogeny and population structure analysis revealed that CIP accessions are typical cultivated autotetraploid potato. A Neighbor-joining tree of all 170 accessions (108 CIP and 62 reported accessions) constructed from random selected 72,923 SNPs from sliding 10-kb window of potato genome showed that analyzed species are mainly divided into Wild, Outgroup, Landrace and Cultivar categories and the CIP accessions belong to Cultivar category. The line colors indicate groups of potato accessions. B Principal component analysis (PCA) revealed that CIP accessions and reported Cultivar are clustered. PC1 mainly divides Wild, Landrace and Cultivar while PC2 mainly divides Landrace, CIP, Outgroup and Wild accessions. C Population structure of 170 accessions by ADMIXTURE showed that 108 CIP accessions belong to Cultivar population. The K values represent the number of clusters. When K = 2, the population were divided into two distinct clusters (Wild species and CIP ones, red and blue respectively); K = 3, the population were divided into Wild (red), Landrace (yellow) and CIP species (blue); K = 4, the population were divided into Wild (red), Landrace (yellow) and Cultivar [including 18 reported North American Cultivars (purple) and CIP species (blue)]; K = 5 to K = 7, reported cultivars were classified into subgroups (green and light blue), the sophisticated genetic structure and missed individuals were shown in CIP cultivars (purple and blue). Each color represents one ancestral population, and each accession is represented by a bar. Length of each colored segment represents the proportion contributed by that ancestral population

Population structure is essential to investigate the background of 108 CIP accessions and an important factor for selective interval analysis and GWAS. According to the phylogeny analysis based on SNP data and the principal-component analysis (PCA), the 170 accessions are mainly divided into five categories including Wild, Outgroup, Landrace, reported cultivar and CIP cultivar (Fig. 1A). Analysis showed a mixture between the CIP cultivars and reported ones, confirming that they both are typical cultivated autotetraploid potato with significant genetic differentiation from Wild and Landrace (Fig. 1A, B).

Variation curve of BIC (Bayesian Information Criterion) value showed the optimal K value, a parameter in K-means clustering used to identify groups and describe the relationship between groups. When K = 2, the population were divided into two distinct clusters only, Wild species and CIP species, while landrace was differentiated when K = 3 or higher. Interestingly, from K = 4, the reported cultivars begun to differentiate from domesticated ones, but a sophisticated genetic structure was observed in CIP cultivars (Fig. 1C, Fig. S1). The differentiation between the reported cultivars and CIP ones may due to that CIP accessions are main cultivated species used for agricultural production with long selection and breeding history than the reported cultivars, and the reported cultivars are mixtures consisting of domestication, improvement, and modern breeding efforts (Hardigan et al. 2017). In addition, differential geographical distribution and genetic distances between them may also result in differentiated population structure considering the relative uniform genetic composition may result from the concentrated geographical distribution (Goulas et al. 2006) and different genetic distances accompanied by unequal wild introgression (Hardigan et al. 2017).

### Domestication analysis dissected the selective sweeps across the whole genome

Domestication of wild potatoes were originated from the Andes of Southern Peru approximately 8000 years ago and were mainly cultivated in altitudes 2000–4000 m regions that are characterized by short day (SD) condition, high light intensities, and cool temperatures (Zierer et al. 2021). Contemporary autotetraploid cultivars were selected to grow under moderate temperatures and to form tubers under long day (LD) condition later (Zierer et al. 2021). We analyzed the phenotypes of 108 CIP accessions which are main germplasm resources with abundant agronomic traits (these accessions are mainly planted in Yunnan or Gansu province of China). Considering the advantage agronomic traits of autotetraploid cultivar tubers are mainly resulted from human selection, identification of the candidate selected loci will shed light on domestication history and breeding improvement of potato.

**Fig. 2:**
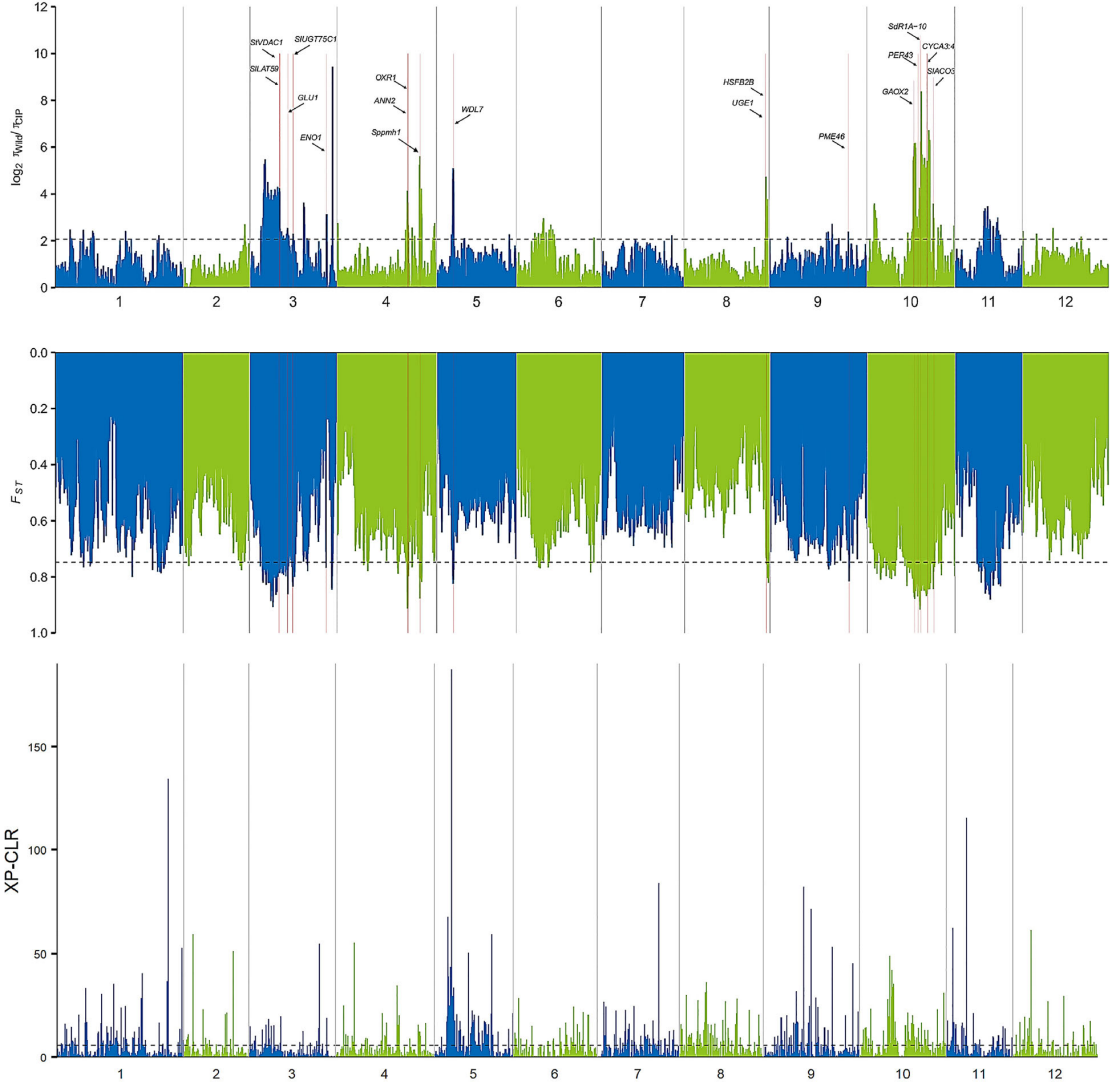
Selective sweep by genome-wide screening and functional annotations during domestication. Selective sweep regions revealed by the log_2_ ratio of nucleotide diversity (Π, upper), Pairwise fixation index (F_ST_, middle) of Wild and CIP, and XP-CLR analysis of CIP and reported cultivars (bottom) on 12 chromosomes. Horizontal dashed lines (log_2_ πWild/πCIP = 2.07, FST = 0.75, or XP-CLR = 1%) indicate the genome-wide threshold of selection signals. The higher the Π value, the greater the difference between two groups. Possible homologous genes of *Arabidopsis* related to domestication traits mentioned in the text were shown at the selected regions of the whole chromosome (upper). Pairwise fixation index (F_ST_) in 20-kb sliding windows across 12 chromosomes revealed the differentiation degree between Wild and CIP subpopulations. The higher the F_ST_ value, the higher degree of differentiation between two subpopulations (middle). XP-CLR scores in 20-kb sliding windows across 12 chromosomes revealed the differentiation between CIP and reported cultivars in the whole genome. The higher the XP-CLR score, the higher degree of differentiation between CIP and reported cultivars (bottom)

To investigate how the genomes of CIP accessions were shaped by environment and human selection, we performed a comprehensive assessment of Wild and CIP population based on genomic analyses of 170 accessions. Selective interval analysis was conducted across the whole genome through a *F*_*ST*_ (population-differentiation statistic) and *θ*_*π*_ (the log2 ratio of nucleotide diversity) based cross approach. As a result, a total of 138 with strong selection signals were identified being involved in various physiological processes including photoperiodic flowering, temperature responses, tuber shape and disease resistance (Fig. 2, Table S5), which may be closely correlated to domestication of cultivars. In addition, based on the identified differentiation between the reported cultivars and CIP ones, an XP-CLR analysis across the whole genome was conducted. XP-CLR scores in 20-kb sliding windows revealed the selective regions and genes in the whole genome (Fig. 2, Table S6). We identified many environmental adaptation related regions and genes including those involve in oxidative phosphorylation and peroxisome process, suggesting that different geographical distribution of these two cultivar groups affected their responses to stress and resulted in differential environmental adaptations. In addition, there are differentiations in the metabolism process-related regions, implying the different potato quality selection and breeding measures by human of two cultivars. However, compared with the result of selective interval analysis between Wild and CIP population, there is little overlap of the selective regions and genes between two groups by XP-CLR analysis, which is in agreement with the population structure divergence analysis with variation curve of BIC since both the reported cultivars and CIP population are cultivated autotetraploid potato with similar genetic distances. Obviously, the differentiation between the two cultivar groups is smaller than Wild and CIP population on account of the long domestication history and breeding improvement of cultivar groups.

Since geographic expansion along latitudinal gradients required an adjustment to different day length and temperature cues (Fuller and Allaby, 2009), gene loci related to photoperiodic response and temperature sensitivity were analyzed by selective interval analysis (Fig. 2). Analysis reveals that *Soltu.DM.10G011640* (homologous to gibberellin oxidase 2, GAox2, in *Arabidopsis thaliana*, Sang et al. 2020) is under strong selection. Studies have proved that tuberization process relies on both GA-dependent and photoperiod-dependent pathways and GA is required for the stolon meristem elongation during the tuberization initiation (Martinez-Garcia et al. 2001; Dutt et al. 2017), and selection of GAox2 is agreement with the essential roles of GA and photoperiod in potato tuberization (Li et al. 2018).

Potato is grown as an annual crop and non-optimal temperature inhibits the growth and survival and hence reduces the tuber yield and productivity (Levy and Veilleux, 2007). Many wild potato species have suffered cool temperatures while moderate temperatures were selected for contemporary autotetraploid cultivars (Zierer et al. 2021). Some temperature sensitivity related genes of potato under selection were identified, including *Soltu.DM.04G021330* and *Soltu.DM.08G027190*, whose homologs of *Arabidopsis* are *ANNAT2* (*ANN2*, Liao et al. 2017) and *HEAT SHOCK TRANSCRIPTION FACTOR B2B* (*HSFB2B*, Charng et al. 2007) respectively, and these genes might be good candidates for breeding improvement of potato in various regions with different temperature preference.

Disease is a big threat to potato yield and quality, and pathogens such as late blight have devastating impacts on potato. Wild potatoes usually possess weaker disease resistance than cultivated potato, and disease resistance is an important agronomic trait under selection analysis. Selective analysis identified some reported potato disease resistant genes including two late blight resistant genes *Soltu.DM.10G012850* (*R1A-10*, Kuang et al. 2005) and *Soltu.DM.10G01398*0 (*SbRGA3*, Song et al. 2003), which will enlighten the potato breeding.

Cultivated potatoes produce bigger tubers than wild potato during domestication of tuber size. Tuber growth is also crucial for yield and productivity after tuberization and genes correlated with cell division were selected. CYCLIN A3;4 (CYCA3;4) is an important protein controlling formative cell divisions in *Arabidopsis* (Willems et al. 2020) and its homolog Soltu.DM.10G014530 was identified. In addition, many stress responsive genes are under selection as well, including microtubule-localized genes *Soltu.DM.03G008530* (*StVDAC1*, Balmer et al. 2004) and *Soltu.DM.05G010550* (*WDL7* in *Arabidopsis*, Dou et al. 2021), which play essential roles in mediating stomatal closure in response to drought stress and ABA treatment. Selection of these genes provides informative hints on the environmental adaptation of potato cultivars and related selective loci can serve as the candidate genetic resources for potato improvement.

### Correlation analysis revealed the close relationship between agronomic traits of CIP accessions

International Potato Center created multiple germplasm resources for variety improvement in different countries. Although their genetic background is mixed without obvious subgrouping, the 108 CIP accessions are core cultivars with a rich diversity among agronomic traits, particularly tuber shape and color (Fig. 4A) as well as differential yield and quality traits which are crucial to the selection of varieties. In addition, the difference of tuber color is the result of metabolism variation. We thus detailed analyzed 25 agronomic traits with statistics of these 108 accessions for several successive years, including 9 yield component traits, 10 quality characters and 6 external properties by planting in Gansu province since 2016 (Table S7).

**Fig. 3:**
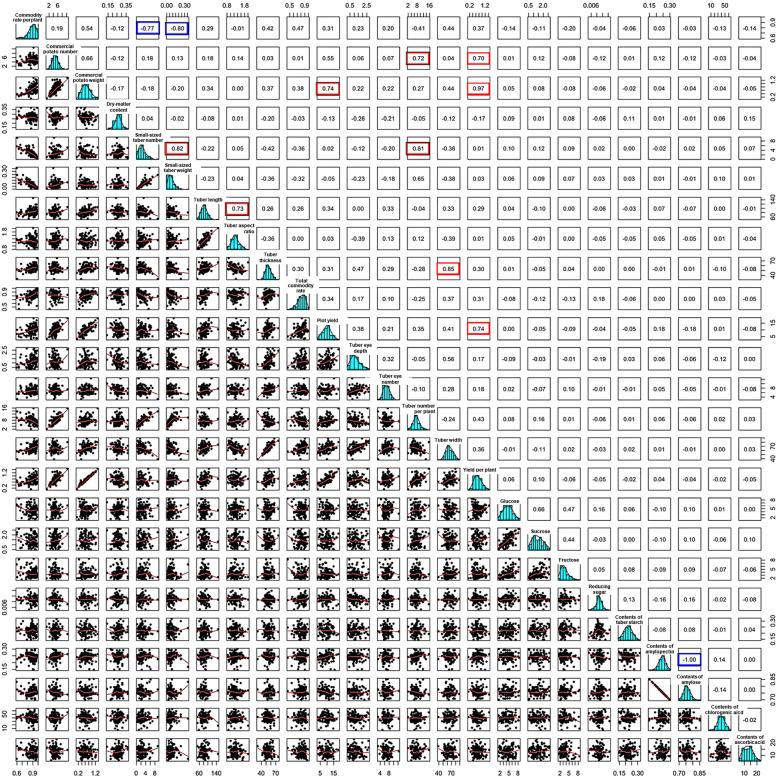
Phenotypic diversity and correlations of 25 traits in CIP potato accessions. Distribution and correlation analysis of 25 agronomic traits showed the normal distribution of each trait. Correlations between different traits are evaluated by Pearson coefficient. The lower left corner is dot graph of two traits, and the upper right corner is the Pearson coefficient between them. For Pearson factor (r), *r* = 0, 1, or − 1 means completely irrelevant, completely positive or negative correlation, respectively. The closer to 1 (or − 1), the stronger the correlation (or negative correlation). Rectangle highlighted by red or blue show strong positive or negative correlation, respectively

Analysis of the analyzed 25 agronomic traits showed that most of them are normally distributed, indicating they are quantitative traits controlled by polygenes and susceptible to environmental influences. Evaluation by Pearson factor further revealed the close correlations of overall 25 traits quantitatively (Fig. 3). Pearson factor evaluation of yield traits including total commodity rate, commodity rate per plant, commercial potato number, commercial potato weight, small-sized tuber number, small-sized tuber weight, tuber number per plant, yield per plant and plot yield, showed the interrelationship between each two of them. In particular, yield per plant is positively correlated to plot yield and commercial potato weight with high Pearson factor (0.74 and 0.97 respectively), indicating the increasing yield per plant is an important means to improve the commercial value of potatoes. All these complex and abundant agronomic traits and their close connections are affected by polygene and environments, which facilitates the genome-wide association analysis to identify QTLs.

**Fig. 4:**
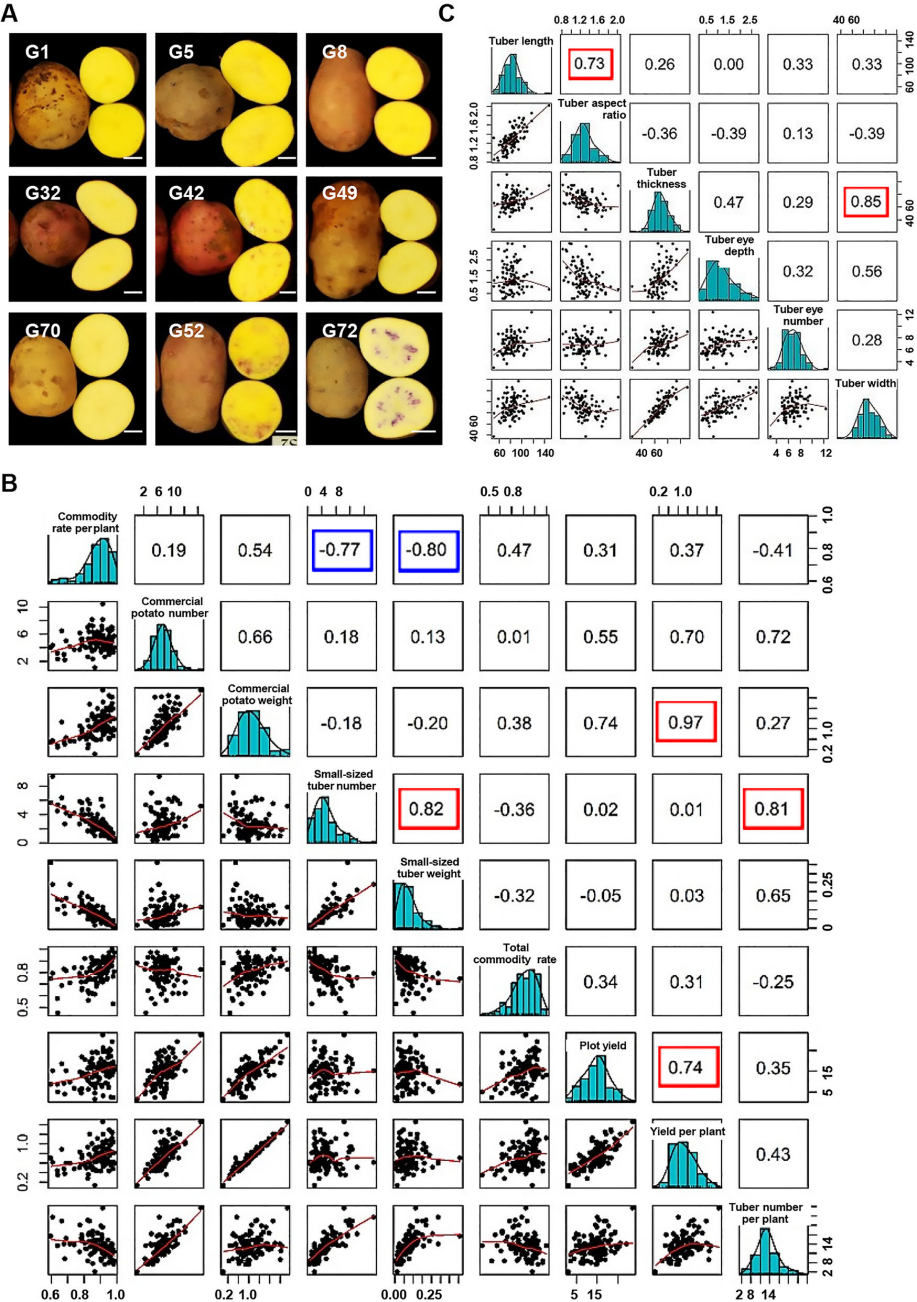
Correlations of the agronomic traits in CIP accessions. A Representative images of potato tuber with different sizes and colors, displaying the diversity of CIP accessions. Bar = 2 cm. B Distribution and correlation analysis of yield traits showed the normal distribution of each trait and close correlations between them evaluated by Pearson factor (r). The lower left corner is the dot graph of two traits, and upper right corner is the Pearson coefficient between them. For Pearson factor (r), *r* = 0, 1 or − 1 means completely irrelevant, completely positive or negative correlation, respectively. The closer to 1 (or − 1), the stronger the positive correlation (or negative). Rectangle highlighted by red or blue show strong positive or negative correlation, respectively. C Distribution and correlation analysis of tuber size related traits showed the normal distribution of each trait and close correlations between them evaluated by Pearson coefficient (r). Tuber size is closely related to traits including length and aspect ratio, tuber thickness and width (highlighted with red rectangle)

**Fig. 5:**
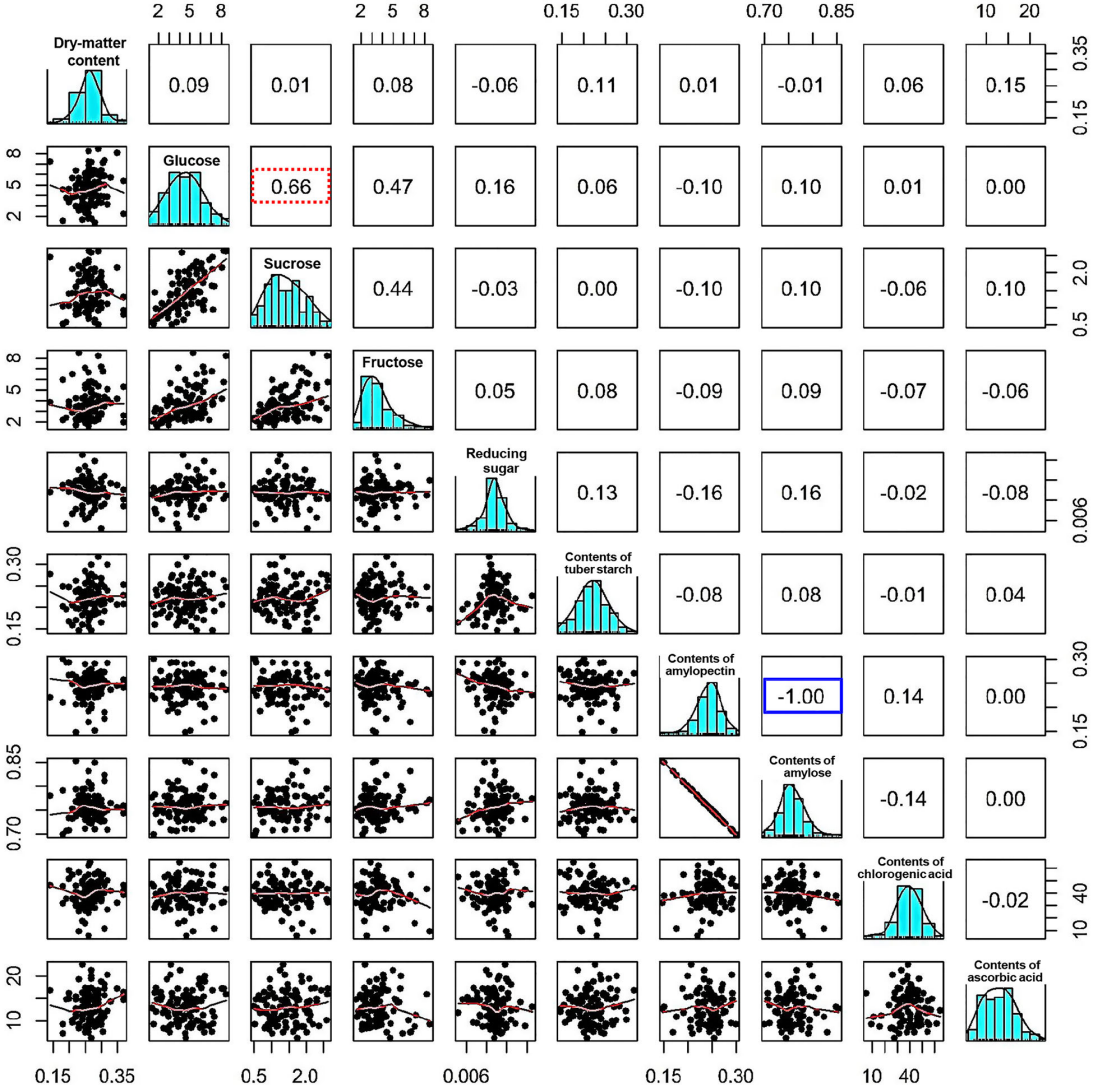
Distributions and correlations of quality traits in CIP accessions. Distribution and correlation analysis of quality traits particularly various sugar contents showed the normal distribution of each trait. Correlations between different traits are evaluated by Pearson factor (r). The lower left corner is the dot graph of two traits, and upper right corner is the Pearson coefficient between them. There is completely negative correlation between contents of amylose and contents of amylopectin (highlighted by blue rectangle) and strong positive correlation between the content of glucose and sucrose (highlighted by red rectangle). For Pearson factor (r), *r* = 0, 1 or − 1 means completely irrelevant, completely positive or negative correlation, respectively. The closer to 1 (or − 1), the stronger the correlation (or negative correlation)

Small-sized tuber number is positively correlated to small-sized tuber weight and tuber number per plant with high Pearson factor (0.82 and 0.81 respectively, Fig. 4B), suggesting increasing tuber number may also increase tuber weight and both are effective ways to improve yield. External properties include tuber length, tuber width, tuber thickness, tuber aspect ratio, tuber’s eye number and the depth of tuber’s eye and all of them are crucial for commercial value and consumers’ acceptance (Si et al. 2017). Close correlation especially tuber size related traits including length and aspect ratio, tuber thickness and width are detected and will enlighten the potato breeding for the larger market demands (Fig. 4C). In addition, examination of the quality traits of dry matter content including tuber starch, contents of amylose and amylopectin, and contents of various sugar and acid showed the great diversity of these traits, revealing the various levels of quality and flavors in CIP accessions. Evaluation by Pearson factor showed a strong negative correlation between the contents of amylose and amylopectin and a positive association between the contents of glucose and sucrose, suggesting the cross of metabolic pathway such as glycometabolism (Fig. 5). The complex quality traits and close correlations also enable the genome-wide association study to reveal the rich and invaluable genetic resource.

### Genome-wide positioned loci associated with key agronomic traits

**Fig. 6:**
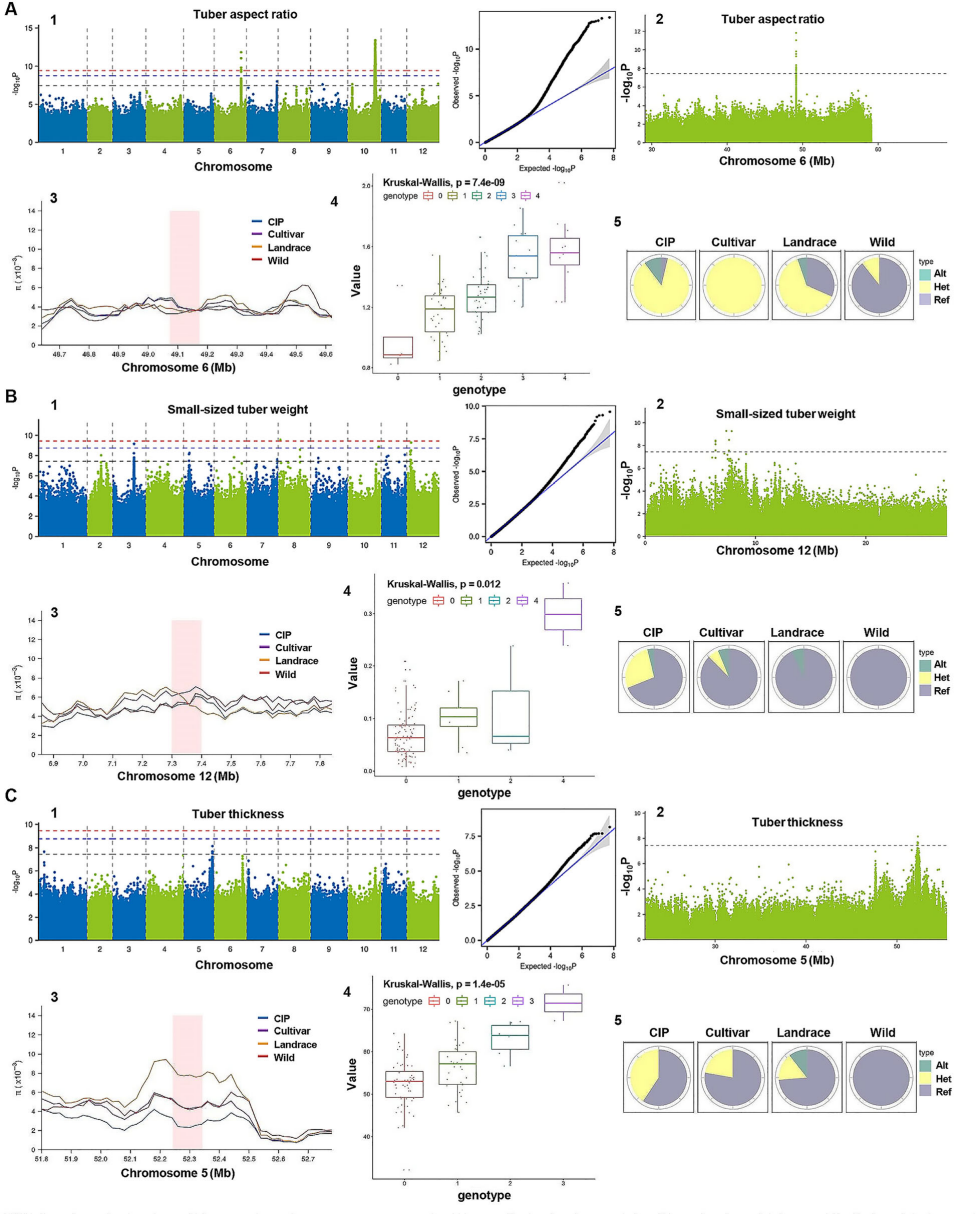
GWAS of potato tuber aspect ratio (**A**), small-sized tuber weight (**B**) and tuber thickness (**C**). 1. Manhattan plot of GWAS (left). The middle horizontal dashed line indicates the genome-wide threshold of GWAS signals with a significance level of 0.05 after Bonferroni correction [0.05/ 25,591,215 = 1.95 × 10^− 9^ (−log_10_P = 8.71)] for multiple tests. The upper and lower horizontal dashed lines mark a significance level of 0.01 and 1, respectively. The corresponding QQ plot (right) showed the distribution of observed *P* values versus those expected under the null for the GWAS. 2. Candidate gene loci by enlarging the region at specific chromosome. The horizontal dashed lines indicate the genome-wide threshold of GWAS signals with a significance level of 0.05. 3. Comparison by the log_10_ ratio of nucleotide diversity (Π) revealed the nucleotide polymorphisms between Wild and CIP populations. 4. SNPs at 3’UTR and its distribution of 108 CIP accessions showed that three examined traits increased significantly with the increase of alternate allele. Numbers of various genotypes were indicated. There are five genotypes in autotetraploid (0, AAAA; 4, aaaa; 1, 2, 3, heterozygotic allele) and three genotypes in diploid (0, AA; 1, Aa; 2, aa). Genotype of reference allele is 0 and alternate allele is 4 (autotetraploid) or 2 (diploid). 5. Genotype distribution of key SNPs in 170 accessions revealed that Wild was dominated by Ref alleles, and Landrace and Cultivar were dominated by Het and Alt genotypes in tuber aspect ratio (**A**); Wild, Landrace and Cultivar was dominated by Ref alleles in small-sized tuber weight (**B**); Wild was dominated by Ref alleles, and Landrace and Cultivar were dominated by Ref and Het genotypes in tuber thickness (**C**). The four pie charts represent the proportion of different genotypes. Genotypes are indicated above (see 4) and reference allele (Ref) is 0, alternate allele (Alt) is 4 (autotetraploid) or 2 (diploid), and heterozygotic allele (Het, 1–3 in autotetraploid or 1 in diploid)

The abundant phenotypic diversity without obvious population differentiation in CIP accessions laid good foundation for GWAS positioning. The variation map at single-base resolution empowered GWAS for 25 agronomic traits in potato. Considering the genome absence of autotetraploid potato and limitation of polyploid GWAS, we applied the GWASpoly to correlate the autotetraploid genotypes for analysis with the data of newly revealed haploid genome (DM v6.1, Pham et al. 2020). In total, 50 association signals containing hundreds of genes related to 15 agronomic traits were identified (Fig. 6, Fig. S2, Fig. S3, Table 1, Table S8), of which some promising loci are consistent with the previous reports.

**Table 1:** The 15 agronomic traits related to yield traits, quality traits and tuber appearance. Candidate loci with association signals were identified by GWAS analysis. Model, the analysis model created with GWASpoly; Chr, the chromosome on which the genes are located; Position, the physical position on chromosome, *P*-value representing the significant difference (−log_10_P)

Trait	Model	Chr	Position	***P***-value
Commercial potato number	additive	chr01	53,546,671	7.751759221
	additive	chr08	9,289,382	7.761336087
Commercial potato weight	additive	chr06	39,598,719	7.635390056
Commodity rate per plant	additive	chr01	1,441,579	8.379762405
	additive	chr05	8,029,132	7.826109398
	additive	chr05	10,378,690	7.722277807
	additive	chr07	20,336,146	7.640000989
Contents of amylopectin	additive	chr01	76,598,419	7.454226593
	additive	chr09	39,683,726	8.201341041
Contents of amylose	additive	chr01	76,598,419	7.454226593
	additive	chr09	39,683,726	8.201341041
Contents of fructose	additive	chr01	1,671,891	11.2415703
	additive	chr04	68,545,453	7.496898574
	additive	chr06	51,907,734	7.494746998
Contents of reducing sugar	additive	chr04	5,121,449	8.068176837
Small-sized tuber number	additive	chr01	46,660,804	8.363685848
	additive	chr08	18,192,967	8.375380861
	additive	chr09	38,612,402	7.756347052
	additive	chr09	65,917,552	7.976222839
	additive	chr12	2,113,621	8.18878601
	additive	chr12	11,738,981	7.646727896
Small-sized tuber weight	additive	chr02	25,319,961	8.039384488
	additive	chr03	39,235,998	9.17556828
	additive	chr04	4,433,795	7.449181657
	additive	chr05	10,344,300	8.275191974
	additive	chr06	36,098,504	7.84763694
	additive	chr07	56,127,633	7.647772326
	additive	chr08	3,879,313	9.571811502
	additive	chr08	39,697,219	8.602640471
	additive	chr09	13,855,548	7.767820029
	additive	chr10	56,486,553	8.87584262
	additive	chr11	8,420,908	7.506651967
	additive	chr11	10,077,049	7.897851041
	additive	chr11	12,782,672	7.98400409
	additive	chr11	43,116,815	8.094322885
	additive	chr12	7,348,885	9.309574598
Total commodity rate	additive	chr07	7,063,854	7.666711254
Tuber aspect ratio	additive	chr06	49,123,341	11.83625679
	additive	chr07	55,669,850	8.023519864
	additive	chr09	21,823,977	7.632673794
	additive	chr10	8,238,500	7.657560212
	additive	chr10	50,375,842	13.41468486
	additive	chr12	57,239,887	7.691485609
Tuber eye depth	additive	chr10	49,591,276	9.441733516
Tuber length	additive	chr09	25,598,280	7.486629211
	additive	chr09	45,761,555	7.474995731
	additive	chr10	50,067,847	7.770368725
Tuber number per plant	additive	chr08	8,407,989	7.809295303
Tuber thickness	additive	chr01	9,983,088	7.677787051
	additive	chr05	52,293,045	8.159705715

Considering the importance of improving tuber yield and quality, tuber aspect ratio and tuber thickness are important traits for tuber shape which may affect the tuber processing and consumer preference, while increasing small-sized tuber weight is the main way to improve the tuber yield and commodity rate. Signals strongly associated with these three traits were further identified and analyzed (Fig. 6).

#### Tuber aspect ratio

A peak strongly associated with aspect ratio on chromosome 10 was identified within the region of previously reported *Ro* (Lozano et al. 2012), a main QTL controlling tuber shape. Nonetheless, there is a lack of *Ro* gene in the haploid reference genome used in this study (Pham et al. 2020) and the appearance of this peak region may due to the copy numbers of *Ro* or adjacent genes. A large peroxidase family on chromosome 10 (from *Soltu.DM.10G018980* to *Soltu.DM.10G019080*) which closely relates to aspect ratio of tuber is dissected as well, which is consistent with that peroxidases play important roles in cell wall elongation as well as in determining shape of potato tubers.

In addition to chromosome 10, a significant GWAS signal on chromosome 6 was identified with many promising loci (Fig. 6, A1). We then analyzed the candidate gene/loci by enlarging this region and determined the accurate loci by population-differentiation statistic (*F*_*ST*_) in 20-kb sliding windows across chromosome 6 (position 48,623,340, Fig. 6, A2). Nucleotide polymorphisms analysis with log_10_ ratio of diversity (Π) showed the insignificant population difference between Wild and CIP populations at this position, indicating the conserved nucleotides during domestication (Fig. 6, A3; Fig. S4A).

Further analysis of SNPs on 3′-UTR and its distribution in 108 CIP populations revealed a significantly increased value of phenotypes with the increase of alternate allele (Fig. 6, A4), suggesting a close correlation between the region and phenotype. Estimation of the genotypic distribution of key SNPs in 170 samples and statistical analysis with Reference Allele (Ref), Alternate allele (Alt), and Heterozygotic Allele (Het) showed that the dominant genotype between Wild and CIP is totally differed (Fig. 6, A5), indicating that key loci in the region might be under selection and domestication.

We next searched the candidate genes mainly based on the report of homologous genes in *Arabidopsis. Soltu.DM.06G023290* encodes gibberellin 20 oxidase 1 (GA20ox1) and affects tuberization and tuber growth by altering GA content, which has been validated in transgenic potato plants (Roumeliotis et al. 2013). Interestingly, GAox family members were also under selection, revealed by the selective analysis. *Soltu.DM.06G022250* encodes protein PHYTOCHROME-DEPENDENT LATE-FLOWERING (PHL) that interacts with PHYTOCHROME B (PHYB) and CONSTANS in *Arabidopsis* (Endo et al. 2013) and may play an role in the long day (LD)-mediated suppression of tuberization and tuber aspect ratio regulation by forming a heterodimer with PHYB (Song et al. 2019). In addition, genes involving in hormone (brassinosteroid, abscisic acid and ethylene) function are also identified, indicated the involvement of hormones in tuber shape regulation (Table S8).

#### Small-sized tuber weight and tuber thickness

Regions strongly associated with small-sized tuber weight were identified on chromosome 12 (Fig. 6, B1) and tuber thickness on chromosome 5 (Fig. 6, C1) respectively, i.e. chromosome 12 (position 6,848,884, Fig. 6, B2) and chromosome 5 (position 51,793,044, Fig. 6, C2) by enlarging the candidate region. Population-differentiation statistic (*F*_*ST*_) and nucleotide polymorphisms analysis with log_10_ ratio of diversity (Π) showed that there is significant population difference between Wild and CIP populations in chromosome 5 associated with tuber thickness and the Wild species signals are significantly higher in this region, indicating a very strong population differentiation relating to domestication (Fig. 6, C3; Fig. S4C), while there is no significant difference in chromosome 12 associated with small-sized tuber weight, suggesting the nucleotide conservation of the loci (Fig. 6, B3; Fig. S4B). Nonetheless, value of both phenotypes increased markedly with the increase of alternate allele, indicating a tight correlation between the loci and phenotypes (Fig. 6, B4; Fig. 6, C4). Further genotypic distribution estimation of the key SNPs showed nearly half dominant genotype of CIP differed with Wild (Fig. 6, B5; Fig. 6, C5), suggesting these two loci might be under selection during domestication.

At the candidate region of chromosome 12, AtOSM34 regulates pathogen response and cell cycle in *Arabidopsis* (Sozzani et al. 2008) and whose homolog in potato was identified from *Soltu.DM.12G007830* to *Soltu.DM.12G007890*, which may affect the small-sized tuber weight by regulating cell division. In addition, *Soltu.DM.12G008030* and *Soltu.DM.12G008040* encode SMALL AUXIN UPREGULATED 66 (SAUR66, Lin et al. 2005) and SAUR20 (Spartz et al. 2012) proteins that involve in auxin effects and regulate sucrose efflux in *Arabidopsis*. Gene *Soltu.DM.12G008510* encodes sugar transporter SWEET7 and involves in sucrose efflux and phloem-mobile tuberization signals (Qu et al. 2012). These genes regulate tuberization as well as tuber growth and development, and may finally affect small-sized tuber weight and yield.

Many hormone related genes, particularly those involving in GA signaling and effects, including *Soltu.DM.05G023410* (*DELLA protein RGA-LIKE 2*, *RGL2*, Gómez et al. 2019) and *Soltu.DM.05G023320* (*gibberellin 2-oxidase 6*) as well as homolog of PHYB *Soltu.DM.05G023390* (Jiang et al. 2020) were selected and identified with association with tuber thickness on chromosome 5.

In addition, key loci associated with other agronomic traits which are closely correlated with potato appearance, yield and quality, and crucial for its commodity value are identified (Fig. S2, Fig. S3, Table 1, Table S8). All associated genes were validated with the expression pattern at different stages of tuber development (Fig. S5) by using tuber transcriptome data (http://solanaceae.plantbiology.msu.edu/pgsc_download.shtml). The identified candidate regions and genes associated with key agronomic traits may be important targets for biotechnological or breeding approaches in the future.

## Discussions

Vegetative propagated crops such as potato are globally important in terms of agricultural production, long-term history of early agriculture and plant domestication as well as planning for more sustainable agricultural futures (Harris, 1972). Domestication represents human-mediated plant evolution revealing a combination of permanent genetic changes and impermanent plastic responses to practices of cultivation or growth environment (Denham et al. 2020). There is still a lack of excellent cultivated varieties with higher yield, better quality and stronger adaptability especially under biotic and abiotic stresses. In addition, the biggest obstacle for molecular research and breeding is the lack of genomic information and identification of gene function due to the analysis difficulty and complexity of autotetraploid genomes. We here reported the resequencing of 108 autotetraploid CIP accessions by whole-genome sequencing and dissected 138 high-confidence selective sweeps comprising 54 predicted genes with selective analysis. In addition, we established and improved the autotetraploid analysis system with GWASpoly software and identified 50 candidate loci associating with 15 agronomic traits related to tuber yield and quality, which provides valuable genomic resources for autotetraploid potato cultivars and lays a good foundation for autotetraploid potato research and further molecular breeding. Identified correlations of diverse agronomic traits not only lay the foundation for elucidating the character elements of potato varieties constitute, properties and the relationship between trait components and total traits, but also help to clarify the process of selection and domestication of common cultivars as well as the GWAS analysis, so as to guide the selection of varieties and potato breeding.

Revelation of genomic sequence at the whole-genome level and identification of functional genes is urgent and essential considering the importance of autotetraploid potato cultivars in product and breeding. Resequencing of autotetraploid CIP accessions with whole-genome sequencing (WGS) fills in blanks of genomic information for autotetraploid cultivars and referenced the newly revealed haploid genome which is a chromosome-scale long-read assembled reference genome (DM v6.1, 16) due to the lack of autotetraploid potato reference genome. Analysis of the diverse agronomic traits is also essential since there are significant differences in yield, resistance and quality, genetic background, ecological adaptability, outward appearance and flavor types. Additionally, 138 high-confidence selective sweeps comprising 54 predicted genes related to flowering time, temperature sensitivity and cell division were also identified by selective interval analysis, which will help to understand the variable traits and complex domestication process of potato adaptation to changed environment and are potential new molecular markers that can be applied for potato breeding. Moreover, the SNPs in candidate genes of GWAS are related to domestication, indicating the changes of key genes in potato selection. Genome variation correlated with domestication revealed how genome reshaped under selection for better phenotypic traits and provides a rich resource for potato cultivation and breeding.

Complex genome with different ploidy of potato and the quantitative nature of most agronomic traits are challenging for accurately understanding the phenotype and genotype relations. GWAS might not be as promising in polyploid species as in diploids since the software tailored to polyploidy is lacking although it has been widely used in diploid species to study the complex traits in diversity and breeding populations. Appropriate GWAS software tailored to polyploidy is the main constraint and biggest challenge to autotetraploid potato species. In this study, we introduced GWASpoly in R, a software tailored for polyploidy and has been utilized in sugarcane and chrysanthemum (Yang et al. 2020; Sumitomo et al. 2019), which could model different types of polyploid gene actions in GWAS, including additive, simplex dominant, and duplex dominant (Rosyara et al. 2016). Combination of GWASpoly and WGS provide more accurate analysis of polyploid genome and may shed light on autotetraploid analysis of other species.

Considering the research combining GWAS with WGS is relative rare in potatoes especially in autotetraploid potato, identification of 50 promising candidate loci including hundreds of genes associating with 15 important agronomic traits of autotetraploid potato by GWAS analysis not only provides new markers for molecular research but also contributes the elucidation of gene effects and functional mechanisms. Although there is still need to further verify the identified loci and genes (gene editing approach will help), identification of them will provide informative clues for potato genetics and breeding practice.

## Methods

### Plant materials and growth condition

A total of 108 potato cultivars in the high generation line catalog of International Potato Center (CIP, https://cipotato.org/catalogue) were analyzed (Supplemental Table 1). These cultivars contain 11 populations from 11 hybridized combinations. The male and female parents of each combination have complex genetic diversity, including Varieties, Population A, BW (bacterial blight resistance), Cycle 0, Cycle 1, Others, B3C1, LBHT-1 (late maturity, late blight resistance, high starch, suitable for processing Fried, heat-resisting), Intermediate LT-LB (medium-early maturity, dishes, late blight resistance), LTVR/LD (adapt to the long day) and LTVR (heat, virus, vegetable, low altitude high temperature adaptability).

Pilot planting was carried out in Weiyuan city, Gansu province in 2016 and 2017. Briefly, a random block design was adopted to sow 50 g cutted potato tuber and each variety has 3 plots whose area was 1.1 m × 2.5 m by single ridge and double row planting with 40 cm row spacing and 25 cm plant spacing. No fertilization and no irrigation were used in all experiments and 10–15 cm depth of soil was covered for seedling consolidation. Phenotypic observation and measurement of various traits were analyzed and evaluated every year.

### Phenotyping

For yield traits, total commodity rate, commodity rate per plant, commercial potato number, commercial potato weight, small-sized tuber number, small-sized tuber weight, tuber number per plant, yield per plant and plot yield were analyzed according to previous study (Zhang and Tian, 2007). For external properties, tuber length (L) was measured from top of tuber to the umbilical cord lengthwise with vernier caliper after washing and drying the tuber. Tuber width (W) was measured horizontally from the widest part of tuber and thickness was measured from the tabletop to the highest point of tuber. Tuber aspect ratio = L/W. The depth of tuber’s eye was measured with a depth ruler, and average number and depth of buds were calculated.

For quality traits, dry matter content was measured by drying weighing method, content of tuber starch was determined by specific gravity method, and contents of amylose and amylopectin by double wavelength method (Zhang and Tian, 2007). Contents of sugar including sucrose, fructose, glucose and reducing sugar, and acid including ascorbic acid, chlorogenic acid were determined according to previously reported method (Ohara-Takada et al. 2005).

### DNA extraction and sequencing

Total genomic DNA was extracted from tuber tissues using the DNeasy Plant Mini Kit (Qiagen). All DNA samples were diluted to 20 ng·μL^− 1^ concentration and stored at − 20 °C. For each accession of CIP panel, at least 5 μg of DNA was used to construct a sequencing library with an Illumina TruSeq DNA Sample Prep Kit according to the manufacturer’s instructions. Paired-end sequencing (150 base pairs) of each library was performed on an Illumina HiSeq X Ten system at Shanghai OEbiotech Co Ltd. (Shanghai, China).

### Reads mapping and variants calling

For the reported 67 accession panel, resequencing data for cultivars, landraces and wild were downloaded from the NCBI database under PRJNA378971 (Hardigan et al. 2017). The raw paired-end reads of 170 accessions were trimmed to remove the adaptors and low-quality bases using FastP (Campos et al. 2006). Reads were filtered with a sliding window of size 4, with average Phred score scale of 20 within the window. Trimmed reads were mapped to the *S. tuberosum* group Phureja DM reference genome (Pham et al. 2020) (Ensembl release 42) using bwamem (Li and Durbin, 2009) (version: 0.7–17) with default parameters. After alignment, Picard tools (version: 2.18.17, http://broadinstitute.github.io/picard/) were used to remove the PCR duplicates according to the mapping coordinates.

Detection of variation is performed following the best practice workflow recommended by GATK (18) (version 3.8.1). In brief, the variants were called for each accession by GATK Haplotype Caller. A joint genotyping step for comprehensive variations union was performed on the gVCF, the ploidy was set 2 or 4 according to the ploidy of accessions. In the filtering step, the SNP filter expression was set as QD < 2.0 || MQ < 20.0 || FS > 60.0 || SOR > 3.0 || MQRankSum < − 12.5 || ReadPosRankSum < − 8.0, and the InDel filter expression was set as QD < 2.0 || FS > 200.0 || MQ < 40.0 || SOR > 10.0 || ReadPosRankSum < − 20.0.

Three subsets of potato SNPs were defined using following filtering criteria: (1) the basic set of 27,565,997 SNPs were created excluding non bi-allelic, > 20% missing calls and MAF < 5%; (2) Phylogeny SNP set of 72,923 SNPs randomly sampled at equal counts from genome-wide 10-kb windows; (3) GWAS SNP set of 27,494,422 SNPs were created by selecting 108 phenotyped CIP individuals by filtering MQ < 40 and MAF < 0.05 form all 170 samples raw vcf.

SNPs and InDels annotation were performed according to the wild castor genome using SNPeff (Cingolan et al. 2012) (version: 4.3 T). The coverage of each accession against each chromosome of grapevine genome was counted based on aligned BAM file using QualiMap (García-Alcalde et al. 2012) (version: 2.2.1) software.

### Population genetics analysis

A phylogenetic tree was constructed from the phylogeny SNP data by using neighbor-joining method in program PHYLIP (Felsenstein, 1989) (version: 3.697, http://evolution.genetics.washington.edu/phylip.html) and colored by R package ggtree (Yu et al. 2017), and IBS distance matrices were calculated using PLINK (Purcell et al. 2007) (version: 1.9). Principal components analysis (PCA) (Price et al. 2006) was performed with EIGENSOFT software. Population structure was analyzed using the ADMIXTURE (Alexander et al. 2009) (version: 1.3) program with a block-relaxation algorithm by collapsed heterozygotes in the dosage model to 0/1 genotypes.

To explore the convergence of individuals, we predefined the number of genetic clusters K from 2 to 10 and ran the cross-validation error (CV) procedure. ADMIXTURE was then run again on the whole core SNP set 10 times with varying random seeds; the Q-matrices were aligned using pong (Behr et al. 2016) software and clustered on the basis of similarity. The matrices belonging to the largest cluster were averaged to produce the final matrix of admixture proportions.

### Genome scanning for selective sweep signals

A genetic differentiation (*F*_*ST*_), nucleotide diversity (*θ*_*π*_) and XP-CLR analysis based cross approach was performed to investigate the selection signals across the whole genome. A 20-kb sliding window with 10-kb step approach was applied to quantify the *F*_*ST*_, *θ*_*π*_ and XP-CLR by using R package PopGenome (Pfeifer et al. 2014). The candidates that meet both top 5% of the *F*_*ST*_ and *θ*_*π*_, and 1% of the XP-CLR were regarded as selective signals.

### Correlation analysis between agronomic traits of CIP accessions

Non-linear regression analysis was performed using R-psych software and presented with Pearson factor. Pearson coefficients were calculated with lm = True (linear regression fits are shown for both y by x and x by y. Correlation ellipses are also shown.)

### Genome-wide association analysis

Since the DAPC analysis showed the best cluster of 108 CIP accessions were 2, we only considered cryptic kinship relationships (K) to minimize false positives and increase statistical power. GWASpoly (Rosyara et al. 2014) is tailored for autopolyploid based on the Q + K mixed model and could model gene actions for polyploids. Six models were used for GWAS by using GWASpoly, including general model, additive model, two simplex dominant models (1-dom-ref and 1-dom-alt), diplo-general model, and diplo-additive model. We defined the whole-genome significance cutoff with the adjusted Bonferroni test threshold, which was set as *P* < 0.05/27,494,422 = 1.82 × 10^− 9^ (−log_10_P = 8.74).

### Transcriptome analysis

FPKM values of all representative transcripts across 40 DM and 16 RH libraries were downloaded from the PGSC data (http://solanaceae.plantbiology.msu.edu/pgsc_download.shtml). Genes in the upstream and downstream 500-kb region of the leading SNPs of each trait were selected and expression pattern associated with the trait was shown except genes whose FPKM equals to 0 across all libraries.

### Supplementary Information

**Additional file 1: Fig. S1.** Cross validation and Bayesian Information Criterion analysis identified the optimal number of subpopulations. **Fig. S2.** Manhattan plot by GWAS for the 12 agronomic traits with association signals. **Fig. S3.** Manhattan plot by GWAS for 10 agronomic traits with weak association signals. **Fig. S4.** Pairwise fixation index (*F*_*ST*_) in potato tuber aspect ratio, small-sized tuber weight and tuber thickness. **Fig. S5.** Transcriptome analysis of the candidate genes by GWAS.

**Additional file 2: Supplemental Table S1.** Sample information for 108 CIP accessions

**Additional file 3: Supplemental Table S2.** Overview of sequencing of samples.

**Additional file 4: Supplemental Table S3.** Statistics of SNPs and InDels for CIP 108 accessions.

**Additional file 5: Supplemental Table S4.** Statistics of SNPs and InDels for 170 accessions.

**Additional file 6: Supplemental Table S5.** Selected intervals and genes between Wild and CIP cultivars.

**Additional file 7: Supplemental Table S6.** Selected intervals and genes between reported and CIP cultivars.

**Additional file 8: Supplemental Table S7.** Phenotypic information of 108 CIP accessions.

**Additional file 9: Supplemental Table S8.** GWAS signals and candidate genes.

## Data Availability

All relevant data are within the manuscript and Supporting information files. Resequencing data of 108 CIP accessions can be found in NCBI database under SUB9867284.

## Abbreviations

GWAS: genome-wide association study
NGS: next-generation sequencing
WGS: whole-genome sequencing
SSR: simple sequence repeat
SNP: Single Nucleotide Polymorphisms
InDel: Insertion and Deletion
BIC: Bayesian Information Criterion
PCA: principal-component analysis
