# Supplementary material for: Resequencing and genome-wide association studies of autotetraploid potato
Source: Mol Hortic. 2022 Feb 10;2:6. doi: 10.1186/s43897-022-00027-y (PMC10515019; doi:10.1186/s43897-022-00027-y)
Supplement: Supplementary file 1 — Additional file 1: Fig. S1. Cross validation and Bayesian Information Criterion analysis identified the optimal number of subpopulations. Fig. S2. Manhattan plot by GWAS for the 12 agronomic traits with association signals. Fig. S3. Manhattan plot by GWAS for 10 agronomic traits with weak association signals. Fig. S4. Pairwise fixation index (FST) in potato tuber aspect ratio, small-sized tuber weight and tuber thickness. Fig. S5. Transcriptome analysis of the candidate genes by GWAS. [file 43897_2022_27_MOESM1_ESM.pdf]

Supplementary Materials include 5 figures and 8 tables and are available online.

Fig. S1. Cross validation and Bayesian Information Criterion analysis identified the optimal number of subpopulations.

Cross validation analysis of population structure showed that optimal K value (K=7) divides the subgroups distinctly.

Fig. S2. Manhattan plot by GWAS for the 12 agronomic traits with association signals.

Manhattan plot of genome-wide association results (left) showed the location of candidate loci on genome of the 12 agronomic traits with association signals besides Figure 3. The middle horizontal dashed line indicates the genome-wide threshold of GWAS signals with a significance level of 0.05 after Bonferroni correction [0.05/ 25,591,215 = 1.95×10^{-9} ($-\log_{10}P=8.71$)] for multiple tests. The upper and lower horizontal dashed lines mark a significance level of 0.01 and 1, respectively. The corresponding QQ plot (right) showed the distribution of observed P values versus those expected under the null for the GWAS.

Fig. S3. Manhattan plot by GWAS for 10 agronomic traits with weak association signals.

Manhattan plot of genome-wide association results (left) showed the location of candidate loci on genome for 10 agronomic traits with weak association signals. The middle horizontal dashed line indicates the genome-wide threshold of GWAS signals with a significance level of 0.05 after Bonferroni correction [0.05/ 25,591,215 = 1.95×10^{-9} ($-\log_{10}P=8.71$)] for multiple tests. The upper and lower horizontal dashed lines mark a significance level of 0.01 and 1, respectively. The corresponding QQ plot (right) showed the distribution of observed P values versus those expected under the null for the GWAS.

Fig. S4. Pairwise fixation index (F_{ST}) in potato tuber aspect ratio, small-sized tuber weight and tuber thickness.

Pairwise fixation index (F_{ST}) in candidate gene of potato tuber aspect ratio (A), small-sized tuber weight (B) and tuber thickness (C) among different potato populations including CIP, Cultivar, Landrace and Wild revealed that Wild has greater

differentiation than CIP populations of candidate gene region.

Fig. S5. Transcriptome analysis of the candidate genes by GWAS.

GWAS identified candidate genes are expressed at different stages during tuber development. Expression pattern of analyzed genes is based on tuber transcriptome data (http://solanaceae.plantbiology.msu.edu/pgsc_download.shtml).

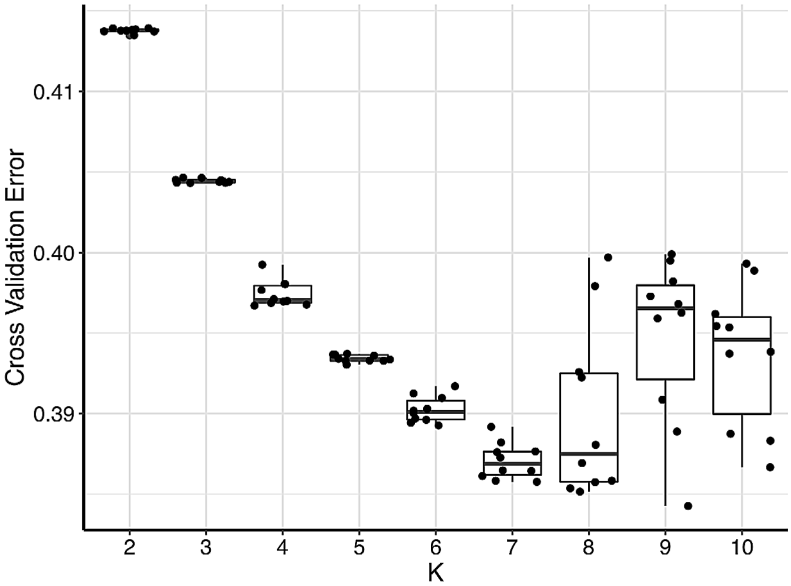

Fig. S1. Cross validation and Bayesian Information Criterion analysis identified the optimal number of subpopulations. Cross validation analysis of population structure showed that optimal K value (K=7) divides the subgroups distinctly.

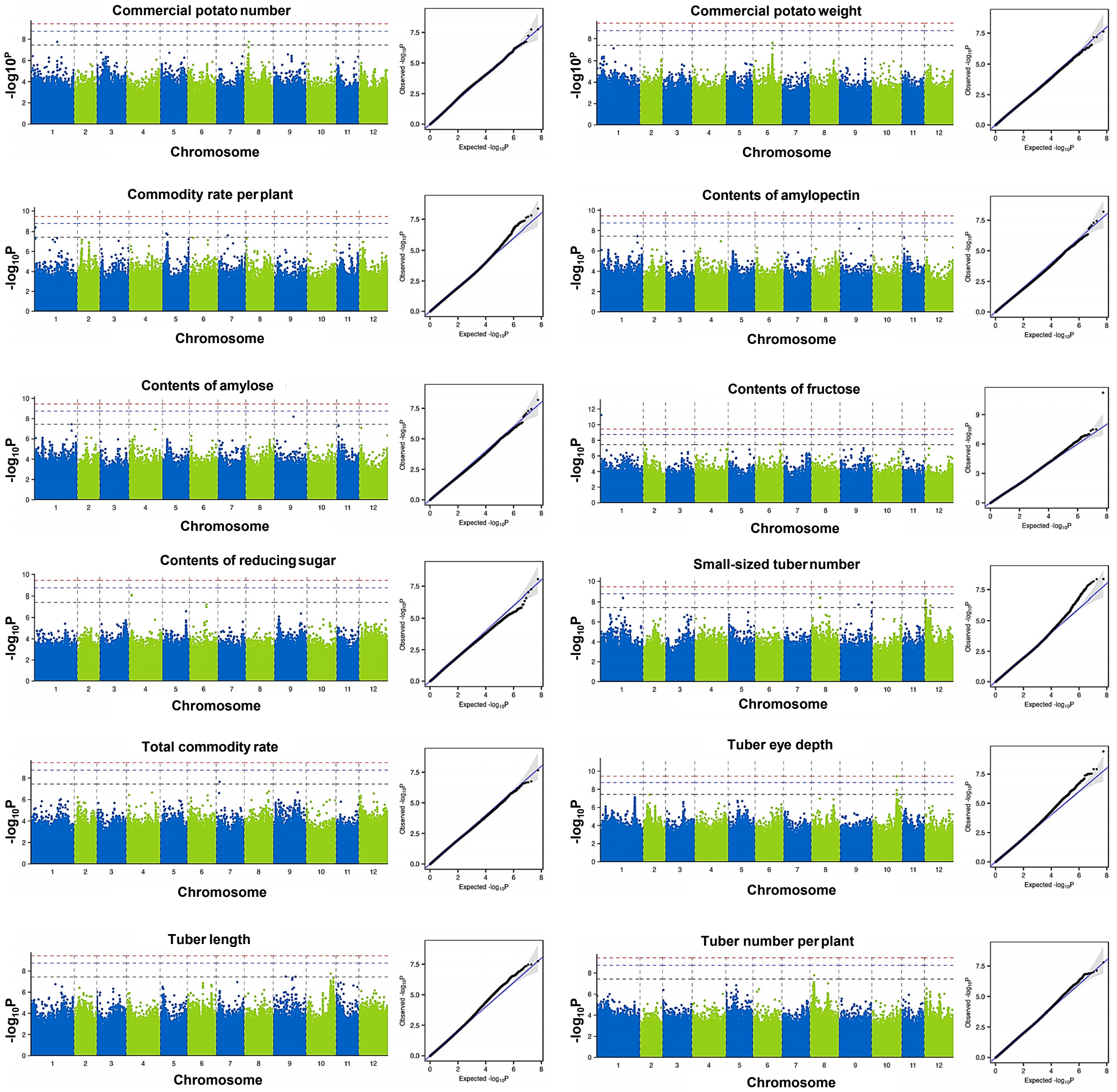

Fig. S2. Manhattan plot by GWAS for the 12 agronomic traits with association signals.

Manhattan plot of genome-wide association results (left) showed the location of candidate loci on genome of the 12 agronomic traits with association signals besides Figure 3. The middle horizontal dashed line indicates the genome-wide threshold of GWAS signals with a significance level of 0.05 after Bonferroni correction [$0.05/ 25,591,215 = 1.95\times10^{-9}$ (-log10P=8.71)] for multiple tests. The upper and lower horizontal dashed lines mark a significance level of 0.01 and 1, respectively. The corresponding QQ plot (right) showed the distribution of observed P values versus those expected under the null for the GWAS.

Zhang et al., Supp Fig 3

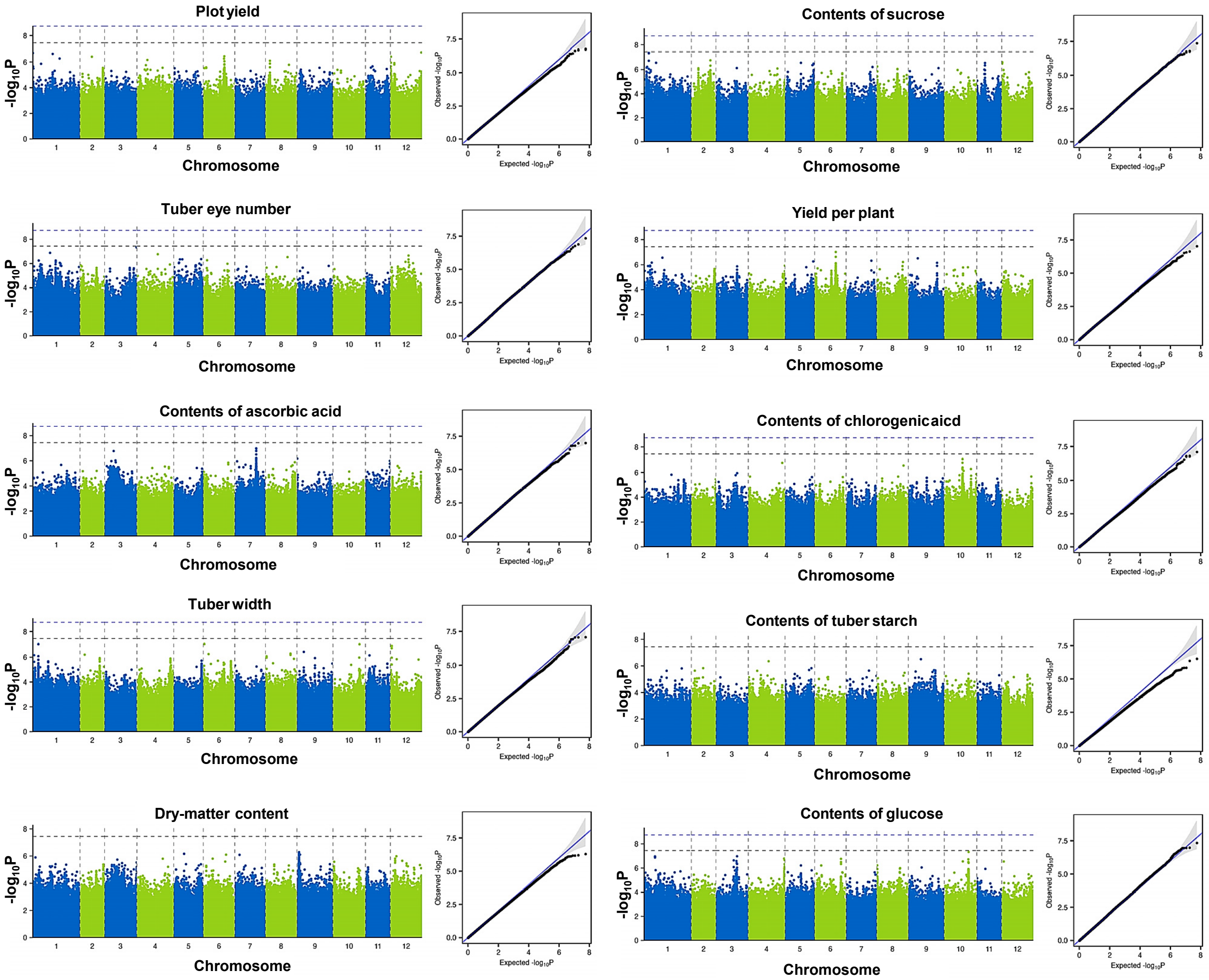

Fig. S3. Manhattan plot by GWAS for 10 agronomic traits with weak association signals.

Manhattan plot of genome-wide association results (left) showed the location of candidate loci on genome for 10 agronomic traits with weak association signals. The middle horizontal dashed line indicates the genome-wide threshold of GWAS signals with a significance level of 0.05 after Bonferroni correction [0.05/ 25,591,215 = 1.95×10^{-9} (-log10P=8.71)] for multiple tests. The upper and lower horizontal dashed lines mark a significance level of 0.01 and 1, respectively. The corresponding QQ plot (right) showed the distribution of observed P values versus those expected under the null for the GWAS.

Zhang et al., Supp Fig 4

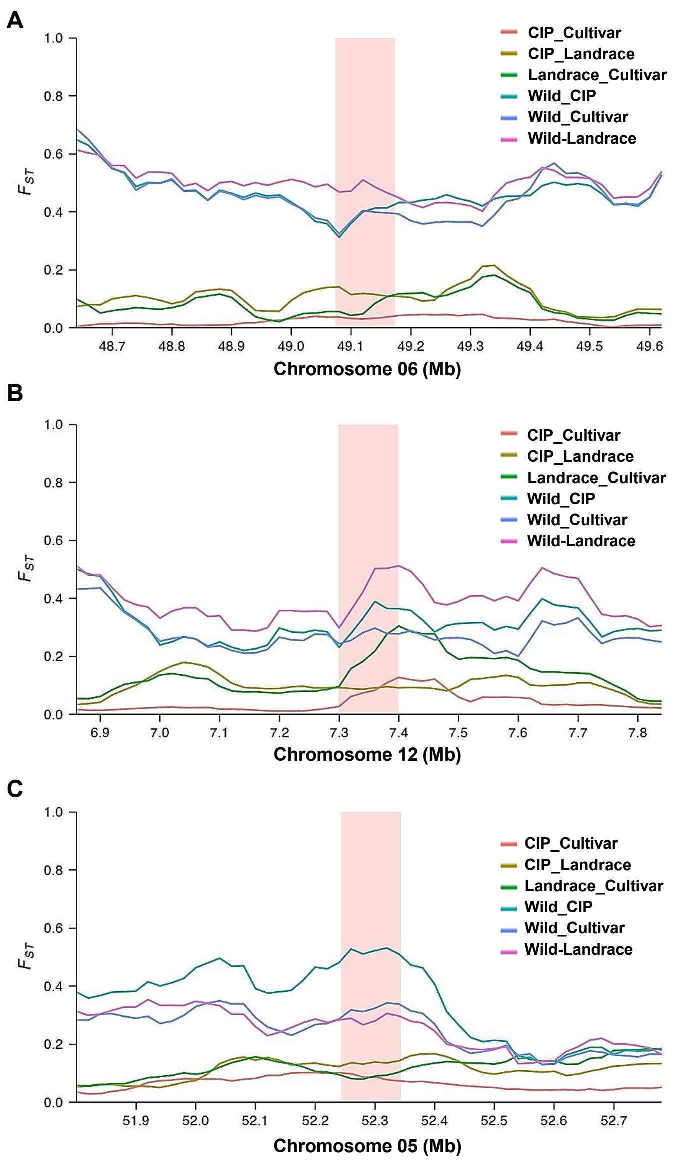

Fig. S4. Pairwise fixation index (F_{ST}) in potato tuber aspect ratio, small-sized tuber weight and tuber thickness.

Pairwise fixation index (F_{ST}) in candidate gene of potato tuber aspect ratio (A), small-sized tuber weight (B) and tuber thickness (C) among different potato populations including CIP, Cultivar, Landrace and Wild revealed that Wild has greater differentiation than CIP populations of candidate gene region.

Zhang et al., Supp Fig 5

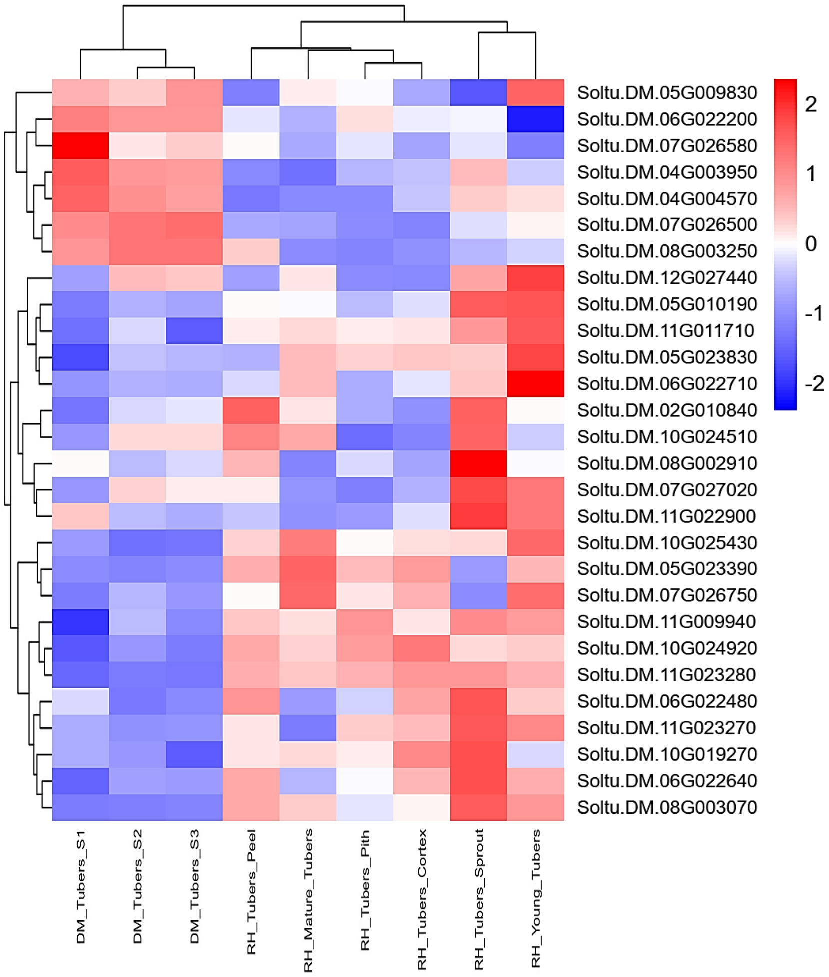

Fig. S5. Transcriptome analysis of the candidate genes by GWAS.

GWAS identified candidate genes are expressed at different stages during tuber development. Expression pattern of analyzed genes is based on tuber transcriptome data (http://solanaceae.plantbiology.msu.edu/pgsc_download.shtml).
